# Septorhinoplasty in sickle cell anemia: a case report^☆^

**DOI:** 10.1016/j.bjorl.2016.08.001

**Published:** 2016-08-23

**Authors:** Can Alper Çağıcı, Süheyl Asma, Mesut Şener

**Affiliations:** a Gazipaşa Mahallesi Baraj Caddesi, Baskent University Adana Seyhan Hospital, Otorhinolaryngology Department, Adana, Turkey; b Gazipaşa Mahallesi Baraj Caddesi, Baskent University Adana Seyhan Hospital, Hematology Department, Adana, Turkey; c Gazipaşa Mahallesi Baraj Caddesi, Baskent University Adana Seyhan Hospital, Anesthesiology Department, Adana, Turkey

## Introduction

Sickle cell anemia is a hereditary disease caused by the presence of hemoglobin S, an abnormal type of hemoglobin. Hemolytic and vaso-occlusive crises are the main manifestations of sickle cell anemia.^1^ Deoxygenation of hemoglobin S may result in intracellular hemoglobin polymerization, which changes the cell morphology and flexibility. The loss of red blood cell flexibility results in occlusion of the capillaries and subsequent vaso-occlusive crises. Vaso-occlusive crises are experienced as severe pain attacks. Recurrent vaso-occlusive crises may result in stroke, renal dysfunction, pulmonary hypertension, retinal disease, and avascular necrosis.^1^, ^2^ Infection, hypoxia, dehydration, acidosis, overexercise, psychological stress, trauma, cocaine use, cold exposure, and high altitude are the predisposing factors to vaso-occlusive crises.^1^ Most of these factors may be seen during general anesthesia and may be controlled by antibiotic prophylaxis, oxygenation, hydration, maintenance of body temperature, and postoperative pain control.^3^, ^4^, ^5^ As with other types of esthetic surgery, rhinoplasty is an elective procedure and might be avoided in this high-risk patient group. This probably explains why we found no reports on this topic in the literature. The current case is believed to be the first esthetic operation in a patient with sickle cell anemia, which makes any surgical procedure requiring anesthesia a high-risk procedure.

## Case report

**Figure 1:**
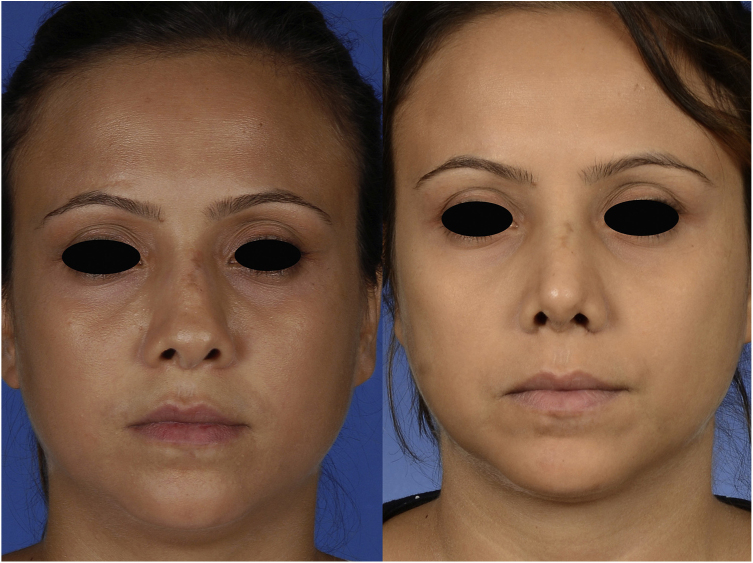
Frontal view of the patient, preoperatively (left) and 1 month postoperatively (right).

**Figure 2:**
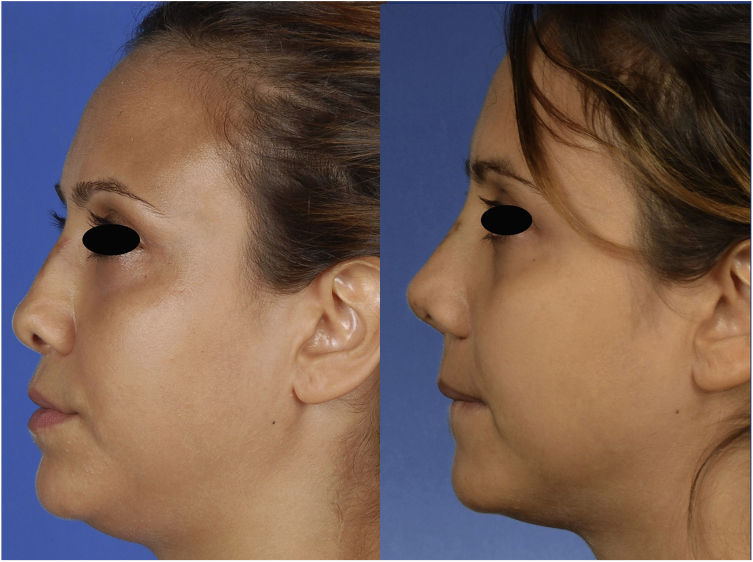
Lateral view of the patient, preoperatively (left) and 1 month postoperatively (right).

**Figure 3:**
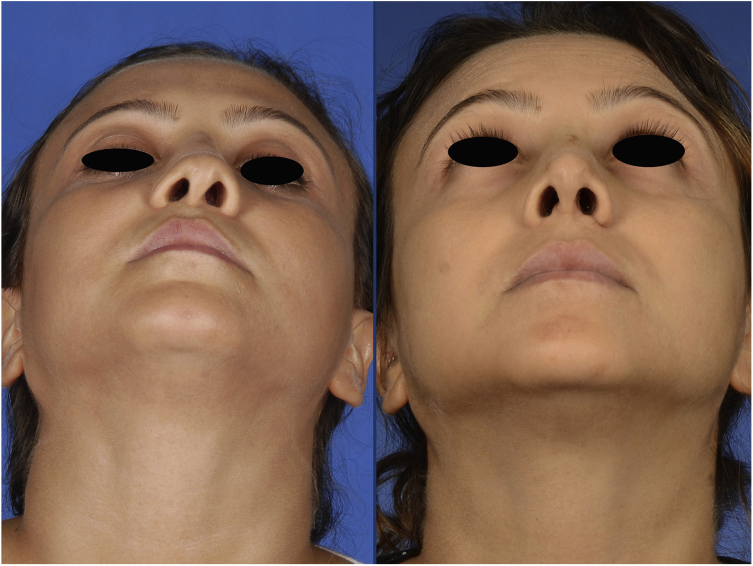
Basal view of the patient, preoperatively (left) and 1 month postoperatively (right).

A 29-year-old woman presented with nasal obstruction. The septum was deviated to the left. Her internal valve was narrow on the left side. She desired to breathe through the nose easily and requested correction of the external deformity (Figure 1, Figure 2, Figure 3) at the time of surgical correction of the septum deviation. She had a history of septoplasty and was being followed by the hematology department because of sickle cell anemia. The patient provided written permission for the publication of her photographs.

Laboratory examination revealed a serum sodium concentration of 138 mEq/L, potassium of 4.81 mEq/L, calcium of 9.19 mg/dL, phosphorus of 3.63 mg/dL, blood urea nitrogen of 10 mg/dL, serum creatinine of 0.44 mg/dL, and uric acid of 3.32 mg/dL. The red cell count was 1.87 × 10^6^ mm^3^, white blood cell count was 5960 mm^3^, hemoglobin concentration was 8.4 g/dL, and hematocrit was 25.7%. Her activated partial thromboplastin time was <26 s, prothrombin time was 13.8 s, and international normalized ratio was 1:1. Her aspartate aminotransferase concentration was 35 IU/L, alanine aminotransferase was 20 IU/L, alkaline phosphatase was 55 IU/L, and γ-glutamyltransferase was 25 IU/L. Her serum lactic dehydrogenase concentration was high at 346 IU/L (normal range, 90–240). Her C-reactive protein concentration was <3 mg/L, and her erythrocyte sedimentation rate was high at 61 mm/h.

The hematology department evaluated the patient preoperatively. We administered two bags of erythrocyte suspension 3 days before surgery, and her hematocrit and hemoglobin concentrations increased from 25.7% and 8.4 g/dL to 32.0% and 11.0 g/dL, respectively. She was evaluated by the anesthesiology department and determined to have an American Society of Anesthesiologists physical status of II.

We entered the septum through a transfixion incision. The septum was deviated to the left. We observed a wide L strut that had been left in place during the previous septoplasty. Although it was sufficient in width, the L strut was not strong enough to support the dorsum and had to be enhanced. We took cartilage grafts from the septum by lifting the intact L strut, which was 1 cm wide. We enhanced the septum by suturing the cartilage grafts to the L strut from the right side.

The lower lateral cartilages were delivered from the infracartilaginous and intercartilaginous incisions. We performed minimal cephalic resection on both sides. We sutured the domes from the cephalic sides. The domes were approximated by interdomal sutures. We required a cap and lateral crural strut grafts and decided to take the grafts from the auricular cartilage. We took a conchal cartilage graft from the right side. The donor side was closed primarily. The cap graft was sutured to the tip using 5/0 polyglycolic acid suture. A lateral crural strut graft was placed under the left lower lateral cartilage.

We rasped the hump and the convexity on the right nasal bone. The cartilage framework was closed using 5–0 polydioxanone sutures. Two layers of crushed cartilage were placed on the supra-tip area. Alar base resection was performed on the left side. Doyle splints were introduced in the nasal passages. An external thermal nasal splint was applied. The operation was terminated without complication.

The septorhinoplasty procedure was performed under general anesthesia. We closely followed the patient's oxygen saturation, a decrease in which may trigger a sickle cell crisis. The minimum oxygen saturation was 99%. The patient was heated perioperatively with a patient-warming system. We did not use epinephrine to avoid a possible vaso-occlusive crisis, and we used only 2% lidocaine hydrochloride as the local anesthetic. Additionally, we did not place an ice bag on the face, which we routinely use in patients undergoing rhinoplasty.

The postoperative period was uneventful without any major hemorrhage. We did not administer additional erythrocyte suspension. On postoperative day 1, the hematocrit and hemoglobin concentration were 25.2% and 8.9 g/dL, respectively. There were no circulation problems in the nasal skin in either the early or late postoperative period. At the time of this writing, the patient was breathing easily through her nose and was happy with her external appearance (Figure 1, Figure 2, Figure 3).

## Discussion

Septorhinoplasty is the most frequently performed esthetic operation. However, there are no data in the literature about esthetic surgery in patients with sickle cell anemia. We performed septorhinoplasty by a closed approach in a patient with sickle cell anemia, and this case is believed to be the first esthetic operation in this patient group. The closed approach is our preference for this procedure.

Hemoglobin S, which is less flexible than normal hemoglobin, causes occlusion of the capillaries and subsequent vaso-occlusive crises. A decreased hemoglobin S concentration and improvement of anemia by preoperative blood transfusion lowers the risk of postoperative complications in patients with sickle cell disease.^6^ Patients with sickle cell anemia may undergo either simple or exchange transfusion. In simple transfusion, the hemoglobin level increases secondary to the blood transfusion. In exchange transfusion, the hemoglobin S is removed from the patient's blood and replaced by a blood transfusion from a healthy individual. The goal of the exchange transfusion is to decrease the hemoglobin S concentration to <30%. The exchange transfusion has the advantages of decreasing the hemoglobin S level without increasing the hematocrit and viscosity of blood.^3^ On the one hand, a Cochrane review reported no difference between simple and exchange transfusion in terms of preventing complications of surgery or sickle cell anemia; however, the review's evidence for this lack of difference was insufficient.^7^ On the other hand, blood transfusion is associated with serious potential complications such as transmission of infection, iron overload, and transfusion reactions.^7^ Although the transfusion may be performed within 2 weeks before surgery, it should be performed 24 h before surgery to maintain a high oxygen transport capacity.^7^

The sickling of erythrocytes in sickle cell anemia results in vaso-occlusive crises, which may cause organ damage and pain. These crises may be triggered by infection, acidosis, trauma, cocaine use, exposure to cold, stress, dehydration, and hypoxia.^1^ Most of these predisposing factors for vaso-occlusive crises may be seen during general anesthesia and must be avoided. Hypothermia, which may result in sickling of erythrocytes, should be avoided. This may be easily achieved by heating of the operation room or patient.^3^ Application of a cold pack to the face, which is routinely applied postoperatively in rhinoplasty, must also be avoided. Perioperative hydration of the patient is important, as is keeping the patient warm. Hypoxia may also trigger sickling. General anesthesia temporarily lowers the oxygen level in the blood, which could be dangerous for patients with sickle cell anemia. Therefore, the oxygen saturation must also be closely monitored. Furthermore, some medications (e.g., decongestants such as pseudoephedrine or epinephrine) can cause vasoconstriction and make it more difficult for the sickle cells to move freely through the blood vessels. A local anesthetic solution that does not contain epinephrine should be used in patients with sickle cell anemia. With proper care, operations under general anesthesia may be safe in patients with sickle cell anemia. Avoidance of dehydration, a low body temperature, and oxygen desaturation as well as the use of a local anesthetic solution without epinephrine should be ensured.^3^ All of these precautions are also valid for esthetic surgery.

Physiologically, it is important to breathe through the nose. Nasal obstruction and breathing through the mouth is not healthy, disturbs the quality of the life, and has to be corrected. Septal deviation is the most frequently seen etiological factor of nasal obstruction, which is corrected by septoplasty. In the present case, the patient had weak septal cartilage that deviated caudally. We corrected the deviation and enhanced the septum using cartilage grafts. The patient also had internal nasal valve collapse, which served as another cause of nasal obstruction. Her left lower cartilage was hypoplastic and corrected with a lateral crural strut graft.^8^ We placed this graft in the submucosal pocket under the lateral crus to support the side wall. Visibility of the cephalic edge of the cartilage graft might be a potential drawback of this technique; however, it was not encountered in the present case, and nasal breathing was successfully achieved.

What is the importance of esthetic surgery in patients with a chronic disease such as sickle cell anemia? Living with a chronic disease can cause anxiety and stress, which often leads to depression. As with cosmetics, feeling more attractive improves the patient's mood, and feeling better helps the patient to live with the chronic disease and increases their quality of life. This is especially important for patients with a chronic disease. Our patient did not seek pure esthetic rhinoplasty. We added esthetic rhinoplasty to the septoplasty operation. Before the present case, we would not normally have accepted a request for pure rhinoplasty from a patient with sickle cell anemia. Our perspective changed after seeing that rhinoplasty was possible in a patient with sickle cell anemia with proper perioperative care, but we are still undecided. On one hand, esthetic surgery is elective and should be avoided in high-risk patient groups. On the other hand, we believe that improving the outlook of patients during management of chronic diseases is as important as treating the disease.

## Conclusion

With proper perioperative care, septorhinoplasty may be performed in patients with sickle cell anemia.

## Ethical approval

This article does not contain any studies with human participants performed by any of the authors.

## Informed consent

Informed consent was obtained from the patient.

## Funding

This research did not receive any specific grant from funding agencies in the public, commercial, or not-for-profit sectors.

## Conflicts of interest

The authors declare no conflicts of interest.
